# Robust facility location in reverse logistics

**DOI:** 10.1007/s10479-021-04405-5

**Published:** 2021-12-02

**Authors:** Péter Egri, Balázs Dávid, Tamás Kis, Miklós Krész

**Affiliations:** 1 Centre of Excellence in Production Informatics and Control, Institute for Computer Science and Control, Budapest, Hungary; 2 Institute for Computer Science and Control, Eötvös Loránd Research Network, Budapest, Hungary; 3 Department of Operations Research, Eötvös Loránd University, Budapest, Hungary; 4 InnoRenew CoE, Izola, Slovenia; 5 University of Primorska, Koper, Slovenia

**Keywords:** Facility location, Robust optimization, Economies of scale, Reverse logistics for wood recycling

## Abstract

As environmental awareness is becoming increasingly important, alternatives are needed for the traditional forward product flows of supply chains. The field of reverse logistics covers activities that aim to recover resources from their final destination, and acts as the foundation of the efficient backward flow of these materials. Designing the appropriate reverse logistics network for a given field is a crucial problem, as this provides the basis for all operations connected to the resource flow. This paper focuses on design questions in the supply network of waste wood, dealing with its collection and transportation to designated processing facilities. The facility location problem is studied for this use-case, and mathematical models are developed that consider economies of scale and the robustness of the problem. A novel approach based on bilevel optimization is used for computing the exact solutions of the robust problem on smaller instances. A local search and a tabu search method is also introduced for solving problems of realistic sizes. The developed models and methods are tested both on real-life and artificial instance sets in order to assess their performance.

## Introduction and motivation

With the recent increase in the importance of environmental awareness, more stress is being put to on the end-of-life recovery and reuse of resources in supply chains. Activities that aim to recover resources from their final destination are integrated by the field of reverse logistics (Dekker et al., 2004). The goal of the reverse logistics is to use these end-of-life resources either to produce further value or to dispose of them properly, usually through a complex recovery process consisting of the stages of repair, reuse, refurbish, remanufacture, retrieve, recycle, incinerate and landfill. Reverse logistics methods can also be integrated into the conventional process of supply chains, forming so-called closed-loop supply chains that account for both forward and reverse flows of resources (Kazemi et al., 2019).

Wood is an extremely versatile raw material with application fields ranging from paper production and packaging to the building industry. Moreover, wooden products can be reused and recycled after their original function becomes obsolete. According to data collected by the Horizon 2020 BioReg Project (Cocchi et al., 2019), the EU countries collectively produced between 40-60 million tonnes of yearly wood waste in the past ten years. Recovery rates of this depend on both the country and the type of wood waste, but it can be seen that there is room for improving the current amounts (Garcia & Hora, 2017).

The amount of research dealing with the management of waste wood has increased over the past years. As an example, the interest can be seen in the furnishing sector, where several studies have been conducted. The paper by de Carvalho Araújo et al. (2019) assesses the literature of circular economy in wood panel production. They conclude that while circular economy as a concept is being investigated with regard to waste production in this sector, mainly LCA (life cycle assessment) studies were carried out (Hossain & Poon, 2018; Kim & Song, 2014). Daian and Ozarska (2009) studied a sample group of six SMEs in the wood furniture sector of Australia and collected data about the current state of their wood waste and its reuse, recycle and disposal. Based on this, they formulated suggestions on wood waste management. Evaluating the availability of wood waste (and wood biomass in general) is also becoming more and more important, which can be seen from the multiple recent studies that have dealt with this question. Research by Verkerk et al. (2019) and Borzecki et al. (2018) assessed the potential availability of forest biomass from European forests and its spatial distribution, focusing on the hotspots of biomass. Studies comparing waste wood management in selected European countries were also conducted by Garcia and Hora (2017) and the BioReg Project (Cocchi et al., 2019).

Although similar studies have become more widespread over the past years, the number of papers dealing with the mathematical modelling and optimization of processes in the waste wood supply chain is still scarce. Network design and planning is one of the most studied problem classes in logistics (Govindan et al., 2015). While there have been recent studies into the combined design of the network nodes and their possible links (Rahmaniani & Ghaderi, 2013), it is usually safe to assume for transport problems that the underlying road network already exists. In this case, the most important problem to solve is facility location. The goal of this problem is to find an optimal placement of facilities on a network in order to minimize arising costs, which usually include transportation and opening facilities. This problem is relevant not only for the forward and reverse supply chains, but also for service industries (Turkoglu & Genevois, 2020).

Mathematical models of facility location are extensively studied, see e.g., Chapter 4 in Dekker et al. (2004). Further variations of the facility location problem (not specific to reverse logistics networks) can be found in Simchi-Levi et al. (2014). Solution approaches includes reformulating the problem as a tree partitioning (Shaw, 1999) and different metaheuristics, such as tabu search (Al-Sultan & Al-Fawzan, 1999).

Stochastic variations of the problem can be found in Verter and Dincer (1992), which also considers capacity planning as the Capacity Expansion Problem once the facility locations are established. Dasci and Laporte study facility location and capacity acquisition by segmenting a market on the infinite continuous plain with uncertain demand (Dasci & Laporte, 2005). In a recent manuscript, Ahmadi-Javid et al. study a combined facility location and capacity planning problem, where the facilities should serve customers with demand modeled as Poisson processes, which results in a nonlinear model (Ahmadi-Javid et al., 2018). Solution methods for facility location with economies of scales are studied in Bucci (2009) and Lu (2010).

Facility location problems usually consider two types of uncertainties; namely, stochastic parameters and disruptions (Peng et al., 2017). An example for the former one is the stochastic demand or cost parameters, see e.g., Carrizosa and Nickel (2003). Robust models, on the other hand considers possible changes in the network structure, e.g., expected consequences of random disruptions or targeted attacks by malevolent attackers (Daskin, 2013). Robust facility location is studied in Cheng et al. (2021).

While general solutions designed for backward biomass streams have been studied in the past [e.g. Nunes et al. (2020), Sharma et al. (2013)], we only found a handful of papers that focus entirely on waste wood. The reverse logistics network redesign problem for waste wood from the construction industry is investigated in Trochu et al. (2018), and a MILP (mixed integer linear programming model) was proposed for its solution. A use-case on a scenario from Quebec, Canada, was also presented. Devjak et al. (1994) formulated a mathematical model for optimizing the transportation of wood waste produced in sawmills, but did not present any computational experiments to back up its efficiency. Burnard et al. (2015) gave a reverse logistics model for facility location and transportation for waste wood, and presented computational results for a use-case in Slovenia.

As it was mentioned before, wood is an extremely versatile raw material, and this property facilitates a wide range of reuse possibilities. While the individual processes of reverse wood supply chains and their order may vary because of differences in regional regulations, a waste wood value chain usually has three major steps: production/collection, sorting/processing and valorization (Cocchi et al., 2019). The origin of waste wood can be manifold, ranging from construction and demolition sites (usually for bulky solid waste) and woodworking industries to waste from households and collection centers (Kharazipour & Kües, 2007). Initially, wood is collected and sorted according to pre-defined quality grades, which will also determine its future use. Higher quality wood is transported for recycle and reuse at processing facilities, producing resources that can compete with freshly harvested wood (Burnard et al., 2015). Waste wood can also be shredded for particle board/wood pellet production, or simply burned for energy (Cocchi et al., 2019). Decontamination of the wood might be needed in certain cases before its processing can start. This is usually done at the same facility as sorting.

As the movement of resources is crucial in this reverse logistics network, the processes connected to collection, transportation and treatment represent a bottleneck in the system (Garcia & Hora, 2017). The recent events of the COVID-19 pandemic showed that this bottleneck is indeed critical, as facility level disruptions caused by reduced staff contributed to the pandemics impact on the supply chain (FAO, 2020). It is pointed out by Ivanov (2020) that the resistance of a supply chain to disruptions is crucial in the case of such extraordinary events, and robustness and recovery should be considered.

In this paper, we consider the facility location problem for transporting waste wood from accumulation centers to processing facilities. Besides transportation, we also study economies of scale as well as the robustness of the network in case of the breakdown of facilities. First, we formulate mathematical models for the problems, including a novel approach based on bilevel optimization, and propose both a local and tabu search heuristic method for their solution. Our model is a special case of the general robust facility location, where any number of facilities can fail. In our special case, simultaneous failure of multiple facilities considered to be extremely rare. This simplification enables the bilevel model to be converted into a series of integer programs that can be solved by standard solvers. To the best of our knowledge, this approach is a novel contribution to the facility location literature. The efficiency of these methods is shown on test instances generated using a real-life dataset as well as different artificial and real-world benchmark datasets from the literature. The conference proceedings article (Egri et al., 2020) presents a preliminary version of this study.

## Problem definition

In the following subsections we formulate the uncapacitated facility location problem and its extensions.

### Uncapacitated facility location problem

Let ${\mathcal {I}}$ denote the set of fixed accumulation point locations and ${\mathcal {J}}$ the set of potential facility locations. Let $f_j$ denote the cost of opening facility *j* and $c_{ij}$ denote the transportation cost from point *i* to facility *j* per m$^3$. Let $u_i$ denote the annual yield of waste wood from accumulation point $i \in {\mathcal {I}}$ (in m$^3$).

The formulation uses two types of binary variables: $Y_j$ is the indicator of opening facility $j \in {\mathcal {J}}$, while $X_{ij}$ indicates product flow from accumulation point *i* to facility *j*. Note that due to uncapacitated facilities, an optimal solution always transports the whole amount of wood from each accumulation point to the closest open facility. The optimization problem is then the following binary integer problem:

1 $$\begin{aligned} \min \quad \sum \limits _{j \in {\mathcal {J}}} f_j Y_j + \sum \limits _{i \in {\mathcal {I}}} \sum \limits _{j \in {\mathcal {J}}} u_i c_{ij} X_{ij} \end{aligned}$$

subject to

2 $$\begin{aligned}&\sum \limits _{j \in {\mathcal {J}}} X_{ij} = 1 \qquad \forall i \in {\mathcal {I}} \end{aligned}$$

3 $$\begin{aligned}&X_{ij} \le Y_j \qquad \forall i \in {{\mathcal {I}}},\ j \in {\mathcal {J}} \end{aligned}$$

4 $$\begin{aligned}&Y_j \in \{\,0,1\,\} \qquad \forall j \in {\mathcal {J}} \end{aligned}$$

5 $$\begin{aligned}&X_{ij} \in \{\,0,1\,\} \qquad \forall i \in {{\mathcal {I}}},\ j \in {\mathcal {J}} \end{aligned}$$

The objective function (1) minimizes the total opening and transportation cost, (2) ensures that the wood is transported from each accumulation point, while (3) states that all wood is transported to an open facility. Constraints (4) and (5) state that the variables are binary.

### Economies of scale problem

It is often realistic to assume that the higher the capacity of a facility, the lower its production cost due to the economies of scale (Garcia & Hora, 2017). We consider the following production cost at facility *j* [based on Bucci (2009)]: $S_j^b p_j$, where $S_j > 0$ is the total quantity processed at facility *j*, $p_j$ is the unit production cost at facility *j* and *b* is a scale factor, typically $-0.35$ for manufacturing facilities and between $-0.56$ and $-0.47$ in the paper industry. With this modification the objective function of the program becomes non-linear as follows:

6 $$\begin{aligned} \min \quad \sum \limits _{j \in {\mathcal {J}}} f_j Y_j + \sum \limits _{i \in {\mathcal {I}}} \sum \limits _{j \in {\mathcal {J}}} u_i c_{ij}X_{ij} + \sum \limits _{j \in {\mathcal {J}}} S_j^b p_j S_j \end{aligned}$$

The constraints are the same as (2)–(5) with the following additional constraint defining the variable $S_j$:

7 $$\begin{aligned} \sum \limits _{i \in {\mathcal {I}}} X_{ij} u_i&= S_j \qquad \forall j \in {\mathcal {J}} \end{aligned}$$

We still consider solutions where wood from each accumulation point is transported to only one facility, since there exist an optimal solution with this property, see Dupont (2008). However, it is no longer true that all wood should necessarily be transported to the closest open facility, for each set of open facilities an assignment problem should be solved to determine the optimal transportation.

### Robust optimization problem

Robust optimization can be modeled as a multi-objective optimization problem, where one objective is minimizing the cost in case of no disruptions, the other is minimizing the cost in case of a disruption. However, we consider only minimizing the cost in case of a disruption instead. More specifically, we consider a solution optimal, if any facility breaks down—i.e., all accumulation points connected with this facility must transport to another facilities—then the resulting cost in the worst case is minimal.

We model this problem as a bilevel optimization: the leader determines which facilities to open, while the follower determines which accumulation point is connected to which facility. The follower’s problem assumes a given set of open and undisrupted facilities ($\{\, j \,|\, Y'_j =1 \,\}$) and assign the accumulation points to these facilities minimizing the transportation costs:

8 $$\begin{aligned} \min \quad \sum \limits _{i \in {\mathcal {I}}} \sum \limits _{j \in {\mathcal {J}}} u_i c_{ij} X_{ij} \end{aligned}$$

subject to

9 $$\begin{aligned}&\sum \limits _{j \in {\mathcal {J}}} X_{ij} = 1 \qquad \forall i \in {\mathcal {I}} \end{aligned}$$

10 $$\begin{aligned}&\sum \limits _{i \in {\mathcal {I}}} X_{ij} \le Y'_j \qquad \forall j \in {\mathcal {J}} \end{aligned}$$

11 $$\begin{aligned}&X_{ij} \in \{\,0,1\,\} \qquad \forall i \in {{\mathcal {I}}}, j \in {\mathcal {J}} \end{aligned}$$

Note that the follower’s problem can be easily solved by transporting all the wood to the closest open facility. Let $G(Y')$ denote the optimal objective value for the follower’s assignment problem on the input vector $Y'$.

Then the leader’s problem is to determine the set of facilities to open with the minimal opening cost together with the transportation cost in case of the disruption of exactly one facility:

12 $$ \begin{aligned} \min _Y \left\{ \sum \limits _{j \in {\mathcal {J}}} f_j Y_j + \max _{Y'} \left\{ G(Y') \,:\, \sum \limits _{j \in {\mathcal {J}}} Y'_j + 1 =\sum \limits _{j \in {\mathcal {J}}} Y_j { \& } Y'_j \le Y_j \ (\forall j \in {\mathcal {J}}) \right\} \right\} \end{aligned}$$

This expresses that facilities $\{\, j \,|\, Y_j =1 \,\}$ are opened, but then one of them cannot be used because of a disruption, therefore the transportation has to be determined not using the disrupted facility. The worst case is considered, i.e., when the disrupted facility causes the highest transportation costs. This corresponds to a pessimistic bilevel program.

## Solution approaches

Solving facility location problems in realistic sizes (i.e., several thousands of accumulation points and possible facility locations) is computationally intractable even without considering economies of scale or robustness. Therefore, similarly to other works in this field, we used metaheuristic algorithms to find quasi-optimal solutions.

### Determining the worst case cost effectively

If economies of scale are disregarded, the optimal solution always transports the wood to the closest open facility. We use this observation to efficiently compute the cost in case of disturbances. Let $\pi _i$ denote a permutation of the facilities for each *i* such that $c_{i \pi _{i1}}< \dots < c_{i \pi _{in}}$, where $n=|{\mathcal {J}}|$ is the number of facilities. If *Y* denote the status of the facilities with at least two open facilities, then let $F_i(Y) = \min \limits _k \{ Y_{\pi _{ik}} = 1 \}$ denote the closest open facility to point *i*, and let $B_i(Y) = \min \limits _k \{ Y_{\pi _{ik}} = 1 \wedge k \ne F_i(Y) \}$ denote the second closest one. If there is a disruption at facility $F_i(Y)$, then the wood from point *i* should be transported to facility $B_i(Y)$ instead, which means $(c_{i B_i(Y)} - c_{i F_i(Y)}) u_i$ additional transportation cost. Therefore in case of a disruption at an open facility *j*, the additional cost is $CoD_j(Y) = \sum \limits _{i : F_i(Y) = j} (c_{i B_i(Y)} - c_{i F_i(Y)}) u_i$. Then the cost increase of disruption in the worst case is simply $\max \limits _{j : Y_j = 1} CoD_j$.

Therefore by maintaining the *F*, *B* and *CoD* vectors when the search heuristics open or close a facility, the value of the objective function can be determined efficiently.

### Local search heuristic

We use the neighborhood defined by Korupolu et al. (2000), which represents the solution only with the set of open facilities. Let $S = \{\, j \,|\, Y_j =1 \,\}$ denote the set of open facilities, then the neighborhood of *S* is $\{\, T \,:\, |S \setminus T| \le 1 \wedge |T \setminus S| \le 1 \,\}$. From a solution *S* one can apply three operations to reach a neighbor: (i) open a new facility, (ii) close a facility (in case $|S>1|$), and (iii) change the status of an open and a closed facility. This neighborhood contains $O(|{{\mathcal {J}}}|^2)$ solutions, where ${\mathcal {J}}$ is the set of potential facilities.

If one intends to solve the robust facility location problem, then instead of the cost defined by (1), the worst case cost should be considered.

### Adaptation of the local search to economies of scale

Considering the economies of scale makes the objective function nonlinear and instead of transporting the wood to the closest open facility, a complex assignment problem has to be solved after determining the set of open facilities. Instead of this, the usual approach is to approximate the objective function with piecewise linear functions. In this section, the basic idea of the linearization and its application for the robust problem is presented.

**Fig. 1:**
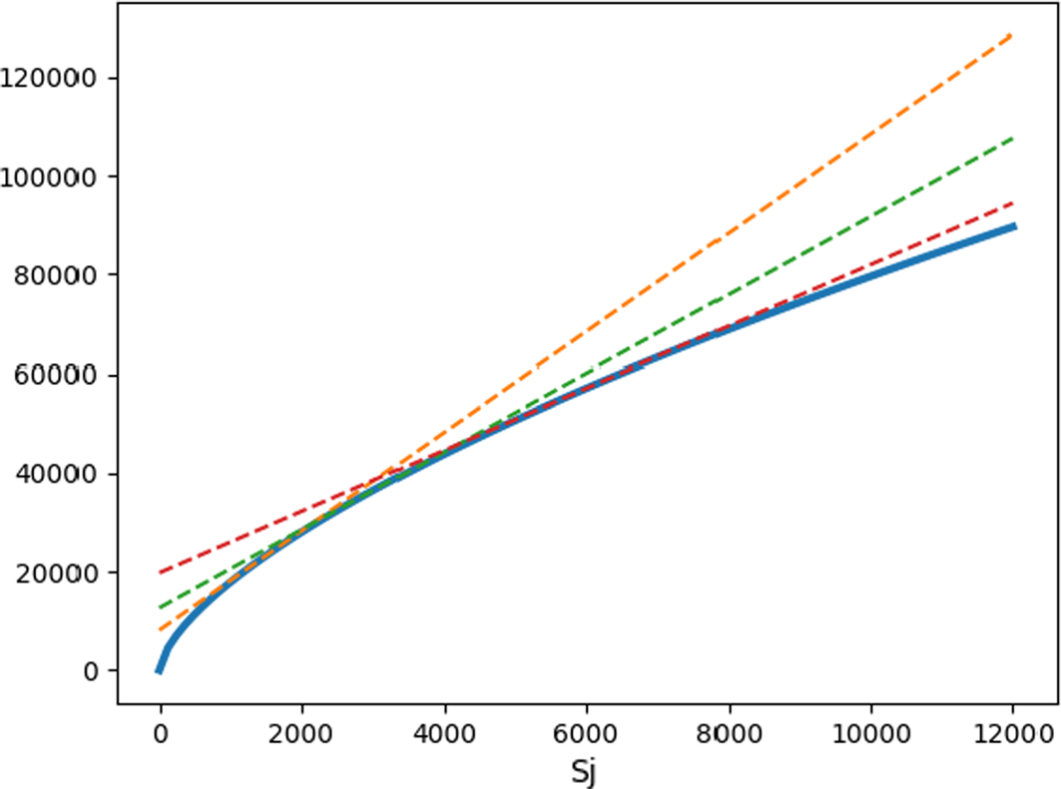
Piecewise linear approximation of the production cost

Figure 1 illustrates a possible linearization of the concave production cost function. The solid line is the $p_j S_j^{b+1}$ production cost (see Sect. 2.2) and the three dashed lines are the tangents at three different points, which provide upper bounds on the production cost. The piecewise linear approximation is then the lower envelope of the tangents. The formula for the tangent touching the concave function at point $s_k$ is

13 $$\begin{aligned} p_j (b+1) s_k^b x - p_j b s_k^{b+1}. \end{aligned}$$

With this linearization, the problem can be reduced to the linear model as follows. Instead of facility *j*, a dummy facility is introduced for each tangent. For the dummy facility representing tangent at $s_k$, the production cost will be the slope of the tangent, i.e., $p_j (b+1) s_k^b$. Furthermore, the opening cost of that dummy facility has to be increased with the amount, where the tangent crosses the y-axis, i.e., the new opening cost will be $f_j - p_j b s_k^{b+1}$. The location of the dummies will be the same as of the real facility, thus the linearization does not affect the transportation costs.

When robustness is not considered, then in an optimal solution only one dummy related to the real factory can be selected at most. Indeed, if multiple dummies are open, then the cost can be decreased by keeping only the one with the smallest production cost open, closing the other dummies and re-assigning their quantities to the remaining open dummy facility. This decreases both the production and the opening costs, while it does not change the transportation cost.

However, this is not automatically true for the robust problem any more. Since a robust solution requires at least two open facilities, a problem with high opening costs might result in a solution with two open dummies, both representing the same facility. In order to avoid this undesired result, the solver has to be modified to explicitly avoid opening more dummies at the same location. In case of the local search heuristic, this means the following modifications of neighborhood: (i) a dummy facility can be opened only if no other dummy facilities are open at the same location, (ii) no modification for the closing operation, and (iii) the status of an open and closed facility can be exchanged only if either these two are at the same location or no open facility exists at the same location as the closed one.

### Tabu search heuristic

We have implemented the tabu search based on the approach described in Sun (2006) with some modifications. In addition to seeking the minimal cost in case of a disruption, we applied a different medium term memory process as well as different approach for updating the lengths of the tabu lists.

The short term memory process is the following. Let *k* denote the number of moves since the start of the search and $\varDelta z^k_j$ the cost change by altering facility *j*’s status, i.e., closing if it is open and open if it is closed. The integer vector ${\mathbf {t}}$ is used to store the last time when the status of the facilities changed, i.e., $t_j$ is the value of *k* when facility *j* changed its status last. Let $z_0$ denote the best objective value in the current search cycle and $k_0$ denote the time when $z_0$ was last updated. Let $z_{00}$ denote the best objective value in the whole search procedure. Let $l_0$ ($l_c$) denote the tabu list sizes for the open (closed) facilities, i.e., they cannot change status twice during this time interval unless the aspiration criterion is satisfied. The aspiration criterion is $z + \varDelta z^k_j < z_0$, where *z* represents the cost of the current solution. This expresses that the status of a facility can be changed if this change results in lower cost than the best objective value of the current cycle. The tabu list sizes are bound by lower limit $l_o^1$ ($l_c^1$) and upper limit $l_o^2$ ($l_c^2$).

Each move is changing the status of a facility. We choose facility ${{\bar{j}}}$, where $\varDelta z^k_{{{\bar{j}}}} = \min \{\, \varDelta z^k_j \,|\, \mathrm {facility}~j~\mathrm {is~not~flagged} \,\}$. A facility ${{\bar{j}}}$ is flagged, if the following tabu condition holds: $k - t_{{{\bar{j}}}} \le l_c$ if $Y_{{{\bar{j}}}} = 0$ or $k - t_{{{\bar{j}}}} \le l_0$ if $Y_{{{\bar{j}}}} = 1$, but does not hold the aspiration criterion. The short term process ends when the solution could not be improved for a specified time, i.e., when $k - k_0 > \alpha _1 n$, where $\alpha _1$ is a parameter of the search.

After each step the lengths of the tabu lists are updated: if the current solution improved the objective value, then $l_0$ ($l_c$) is increased by one, otherwise it is decreased by one to extend the search space.

In the medium term, we changed the frequency based memory process described by Sun (2006) and use a wider neighborhood instead. We seek for an open and a closed facility such that if we change their statuses, the total cost decreases the most. Sun states that considering this operation is costly, but our algorithm only use it when the short term process fails to improve the solution, thus providing a trade-off between computation time and solution quality. We have found that this approach performs better on the tested instances.

If the solution can be improved, the search continues with the short term process. The medium term process ends when no improvement can be found.

Finally, the long term process is invoked *C* times and when invoking the *c*th time, *c* moves are made changing the status of facility ${{\bar{j}}}$ according to the following criterion: $t_{{{\bar{j}}}} = \min \{\, t_j \,|\, j=1,\dots ,n \,\}$.

Let ${\bar{S}} = \{\, j \,|\, Y_j =0 \,\}$ and $S = \{\, j \,|\, Y_j =1 \,\}$ denote the indices of the open and closed facilities, respectively. The following is a step-by-step description of the procedure, based on Sun (2006). Note that the main differences are in steps 5–7 that describe the medium term process.

*Initialization*

- *Step 0.* Find a local optimal solution with a greedy method. Let *z* represent the objective value of the current solution. Let $z_0\leftarrow z$ and $z_{00}\leftarrow z$. Select values for $l_c^1$, $l_c^2$, $l_o^1$ and $l_o^2$ and determine the initial tabu sizes $l_c$ and $l_o$ such that $l_c^1\le l_c\le l_c^2$ and $l_o^1\le l_o\le l_o^2$. Let $t_j\leftarrow -l_c$ for all $j\in \bar{S}$ and $t_j\leftarrow -l_o$ for all $j\in S$ to initialize the vector **t**. Select values for $\alpha _1, \alpha _2$ and *C*. Let $k\leftarrow 1, k_0\leftarrow 1, c_1\leftarrow 1$ and $c\leftarrow 1$. Compute the $\varDelta z_j^1$ values.

*Short term process steps*

- *Step 1.* Select a facility $\bar{j}$, where $\varDelta z^k_{\bar{j}} = \min \{\, \varDelta z^k_j \,|\, \mathrm {facility}~j~\mathrm {is~not~flagged} \,\}$. Check the tabu status of the selected move. If tabu, go to Step 2; otherwise, go to Step 3.
- *Step 2.* Check the aspiration criterion of the selected move. If satisfied, go to Step 3; otherwise, mark facility $\bar{j}$ as flagged and go to Step 1.
- *Step 3.* Let $y_{\bar{j}}\leftarrow 1 - y_{\bar{j}}$, $z\leftarrow z + \varDelta z^k_{\bar{j}}$, $t_{\bar{j}}\leftarrow k$ and $k\leftarrow k+1$. If $z<z_0$, let $z_0\leftarrow z$ and $k_0\leftarrow k$. If $z<z_{00}$, let $z_{00}\leftarrow z$. If $\varDelta z^k_{\bar{j}} < 0$, i.e., the change improved the cost, increase the length of the tabu list by one ($l_c$ or $l_o$, depending on whether an opening or a closing operation has been performed), otherwise decrease the length by one.
- *Step 4.* Update $\varDelta z^k_j$. Mark each facility *j* as unflagged. If $k-k_0\le \alpha _1 n$, go to Step 1; if $k-k_0\le (\alpha _1 + \alpha _2) n$, continue to Step 5; otherwise, go to Step 8.

*Medium term process steps*

- *Step 5.* Select $\bar{j}_1 \in \bar{S}$ and $\bar{j}_2 \in S$, such that simultaneously opening $\bar{j}_1$ and closing $\bar{j}_2$ results in the minimal cost. If this cost is not less than the cost of the current solution, go to Step 8.
- *Step 6.* Open facility $\bar{j}_1$ and close facility $\bar{j}_2$. Let $t_{\bar{j}_1}\leftarrow k$, $t_{\bar{j}_2}\leftarrow k$ and $k\leftarrow k+1$. Compute the *z* objective value.
- *Step 7.* If $z<z_0$, let $z_0\leftarrow z$ and $k_0\leftarrow k$. If $z<z_{00}$, let $z_{00}\leftarrow z$. Go to Step 4.

*Long term process steps*

- *Step 8.* If a local optimal solution has not been found, select a facility $\bar{j}$, where $\varDelta z^k_{\bar{j}} = \min \{\, \varDelta z^k_j \,|\, \mathrm {facility}~j~\mathrm {is~not~flagged} \,\}$ and go to Step 3.
- *Step 9.* If $c>C$, Stop. If $c_0>c$ go to Step 11.
- *Step 10.* Let $c_0\leftarrow c_0+1$. Select a facility $\bar{j}$, where $t_{\bar{j}} = \min \{\, t_j \,|\, j=1,\dots ,n \,\}$ and go to Step 3.
- *Step 11.* Let $c_0\leftarrow 1, c\leftarrow c+1, z_0\leftarrow z$, and $k_0\leftarrow k$. Reset the value of $l_c$ and $l_o$ such that $l_c^1\le l_c\le l_c^2$ and $l_o^1\le l_o\le l_o^2$ and go to Step 4.

### Bilevel integer program formulation

Considering the formulation of Sect. 2.3, it can be observed that once the $Y'$ variables are fixed, the *X* variables are easy to determine to minimize the transportation costs by assigning each accumulation point to the closest open facility. This suggests that a solution of the following constraints determines an optimal assignment.

14 $$\begin{aligned}&X_{i\pi _{i1}} \ge Y'_{\pi _{i1}} \qquad \forall i \in \mathcal {I} \end{aligned}$$

15 $$\begin{aligned}&X_{i\pi _{ik}} \ge Y'_{\pi _{ik}} - \sum _{t=1}^{k-1} Y'_{\pi _{it}} \qquad \forall i\in \mathcal {I}, k=2,\ldots ,n \end{aligned}$$

16 $$\begin{aligned}&\sum _{j\in \mathcal {J}} X_{ij} = 1 \qquad \forall i\in \mathcal {I}\end{aligned}$$

17 $$\begin{aligned}&X_{ij} \in \{\,0,1\,\} \qquad \forall i\in \mathcal {I}, j \in {\mathcal {J}} \end{aligned}$$

Then, the inner maximization problem of (12) takes the form

18 $$\begin{aligned} \max \sum \limits _{i \in {\mathcal {I}}} \sum \limits _{j \in {\mathcal {J}}} u_i c_{ij} X_{ij} \end{aligned}$$

subject to (14)–(17) and the constraints

19 $$\begin{aligned}&\sum \limits _{j \in {\mathcal {J}}} Y'_j + 1 = \sum \limits _{j \in {\mathcal {J}}} Y_j \end{aligned}$$

20 $$\begin{aligned}&Y'_j \le Y_j \qquad \forall j \in {\mathcal {J}} \end{aligned}$$

Note that this formulation does not include non-linearity in contrast to the usual duality-based formulation [see e.g., Cheng et al. (2021)].

Using this observation, we search for the optimal solution where exactly *k* facilities ($\rho _1< \cdots < \rho _k$) are open: $\sum _{j \in {\mathcal {J}}} Y_j = k$ and $Y_{\rho _l} = 1$ ($l \in \{\, 1, \dots , k \,\}$). Let $Y^l$ denote the vector that differs from *Y* only in its $\rho _l$th element and $\{\, X_{ij}^l \,:\, i \in {{\mathcal {I}}}, j \in {{\mathcal {J}}} \,\}$ the optimal transportation from accumulation point *i* to facility *j* using open factories determined by $Y^l$. Then the optimization problem (12) becomes:

21 $$\begin{aligned} \min \quad \sum \limits _{j \in {\mathcal {J}}} f_j Y_j + z \end{aligned}$$

subject to

22 $$\begin{aligned}&z \ge \sum _{i \in \mathcal {I},j \in \mathcal {J}} u_i c_{ij} X_{ij}^l \qquad \forall l \in \{\, 1, \dots , k \,\} \end{aligned}$$

23 $$\begin{aligned}&X_{i\pi _{i1}}^l \ge Y^l_{\pi _{i1}} \qquad \forall i \in \mathcal {I}, l \in \{\, 1, \dots , k \,\} \end{aligned}$$

24 $$\begin{aligned}&X_{i\pi _{is}}^l \ge Y_{\pi _{is}}^l - \sum _{t=1}^{s-1} Y_{\pi _{it}}^l \qquad \forall i\in \mathcal {I}, s=2,\ldots ,n, l \in \{\, 1, \dots , k \,\} \end{aligned}$$

25 $$\begin{aligned}&\sum _{j\in \mathcal {J}} X_{ij}^l = 1 \qquad \forall i\in \mathcal {I}, l \in \{\, 1, \dots , k \,\} \end{aligned}$$

26 $$\begin{aligned}&\sum _{j \in {\mathcal {J}}} Y^l_j = k-1 \qquad \forall l \in \{\, 1, \dots , k \,\} \end{aligned}$$

27 $$\begin{aligned}&Y^l_j \le Y_j \qquad \forall j\in \mathcal {J}, l \in \{\, 1, \dots , k \,\} \end{aligned}$$

28 $$\begin{aligned}&\sum _{l=1}^k Y^l_j = (k-1)Y_j, \qquad \forall j \in \mathcal {J} \end{aligned}$$

29 $$\begin{aligned}&\sum _{j \in {\mathcal {J}}} Y_j = k \end{aligned}$$

30 $$\begin{aligned}&Y_j \in \{\,0,1\,\} \qquad \forall j \in {\mathcal {J}} \end{aligned}$$

31 $$\begin{aligned}&Y^l_j \in \{\,0,1\,\} \qquad \forall j \in {{\mathcal {J}}}, l \in \{\, 1, \dots , k \,\} \end{aligned}$$

32 $$\begin{aligned}&X_{ij}^l \in \{\,0,1\,\} \qquad \forall i\in \mathcal {I}, j \in {{\mathcal {J}}}, l \in \{\, 1, \dots , k \,\} \end{aligned}$$

Constraints (23)–(25) are the constraints of the inner optimization problem. Inequality (28) says that if $Y_j = 0$, then all $Y^l_j=0$, whereas if $Y_j = 1$, then exactly $k-1$ of the $Y^l$ has a 1 in position *j*. This, along with (26) and (27) implies that vectors $Y^l$ are all different, they are not bigger than *Y* (coordinatewise), and they have $k-1$ coordinates of value 1, all other coordinates being 0.

This formulation considers a fixed number of open facilities, therefore it should be solved for all possible (or realistic) *k* values.

## Numerical study

The efficiency of the bilevel formulation as well as of the local and tabu search heuristics were tested on several instance sets of different structure and origin. While a detailed analysis was performed on an industrial dataset of Austrian accumulation points, we also examined benchmark datasets and other real-world dataset found in the literature. The results of these tests can be seen in the following sections.

The experiments were run on a standard laptop computer with Intel Core i7 processor. The runtimes of the different algorithms depend on several factors, including the size of the problem and the cost parameters. For example, the binary integer programming formulation of a 500 location problem was solved on average in 15 seconds when the facility opening cost was 5 million, 8 seconds when the opening cost was set to 500,000, and further decreased to 3–4 s with 50,000 as the opening cost. For the same instances, the local search ran in 10 seconds in the first case, but the runtime went up to several minutes when the opening cost was decreased to 50,000. The reason for this is the reduction in the size of the search space of the local search in the case of unrealistically large opening cost, as the optimal solution opens only a few facilities. Finally, the runtime of the tabu search is the largest, usually over 10 minutes. In this case, the runtime depends mainly on $\alpha _1, \alpha _2$ and *C*, which determine the length of the search, even if the algorithm finds an optimal or local optimal solution. Since the robust facility location model with economies of scale presented in this paper differs from the previous models in the literature, it would not be reasonable to directly compare the presented algorithms with other approaches in the literature.

### Numerical study of the Austrian network

Based on the industrial dataset of 1839 accumulation points and possible facility locations, we generated test sets containing 50, 100 and 500 locations, five different test cases for each set. Then we computed the solutions assuming different facility opening costs from the realistic 5 million to 1000. The solutions were computed using the local search, the tabu search, and when possible, the exact solver. For the tabu search we used the same parameters as Sun (2006): $l_c^1 = l_o^1 = 10$, $l_c^2 = l_o^2 = 20$, $C=5$ and $\alpha _1 = 2.5$.

Table 1 contains the average results over the five test sets. The non-robust solutions aim at minimizing the total opening and transportation cost indicated in the cost column, while robust solutions aim at minimizing the worst case cost (WCC), i.e., the total opening cost and transportation costs in case of a facility disruption.

**Table 1:** Average performance using 50 locations

Opening	Non-robust			Robust					
	Open	Cost	WCC	Open	Cost	WCC	CoD (%)	PoR (%)	BoR (%)
5,000,000 (OPT)	1	6,452,010	–	2	11,349,166	11,494,142	1.28	75.98	–
5,000,000 (LS/TS)	1	6,452,010	–	2	11,349,166	11,494,142	1.28	75.98	–
2,500,000 (OPT)	1	3,952,010	–	2	6,349,166	6,494,142	2.31	60.81	–
2,500,000 (LS/TS)	1	3,952,010	-	2	6,349,166	6,494,142	2.31	60.81	–
1,666,666 (OPT)	1	3,118,677	–	2	4,682,499	4,827,476	3.14	50.34	–
1,666,666 (LS/TS)	1	3,118,677	–	2	4,682,499	4,827,476	3.14	50.34	–
1,000,000 (OPT)	1	2,452,010	–	2	3,349,166	3,494,142	4.42	36.81	–
1,000,000 (LS/TS)	1	2,452,010	–	2	3,349,166	3,494,142	4.42	36.81	–
1000 (OPT)	40.8	46,360	102,481						
1000 (LS)	40.6	46,377	102,498	41.4	46,534	93,605	102.20	0.34	10.57
1000 (TS)	40.8	46,360	102,481	41.6	46,518	93,588	102.21	0.34	10.58

We have estimated the production cost for the facility location model with the economies of scale, however, we have found that for realistic cases (large number of accumulation points, large facility opening costs, few open facilities) the economies of scale does not influence the solution (see Sect. 4.3). Therefore the non-linearity of the problem was not considered in the results presented below, which resulted in more tractable problems.

The following indicators are included in the table: the cost of disruption (CoD) is the additional cost in case of a disturbance [(WCC-cost)/cost], the price of robustness (PoR) is the difference between the robust solution and the non-robust one [(robust cost − non-robust cost)/non-robust cost] and the benefit of robustness (BoR) is the difference in case of a disruption [(non-robust WCC − robust WCC)/non-robust WCC]. This latter indicator cannot be interpreted when the non-robust solution contains only one opened facility, i.e., when in case of a disruption the whole network fails.

The rows labelled with “OPT” denote the average costs of the optimal solutions. For the non-robust problem, it is computed by the the FICO XPRESS Solver using the formulation in Sect. 2.1, and for the robust problem the optimum is computed by solving the bilevel programming formulation of Sect. 3.5.

Table 1 contains the results of the solutions considering 50 locations. The following observations were made:

- For the opening costs between 1 million and 5 million, the exact solutions could be computed for the non-robust, and the robust variants as well, and both the local search and the tabu search could find the optimal solution in every case.
- Changing the opening costs in a wide range (above one million) did not change the solutions. That means that the uncertainty of the exact opening cost does not matter too much.
- For the 4 largest facility opening costs, the non-robust solutions contain only one opened facility, therefore they are quite vulnerable for disruptions. Adding one more facility to improve robustness is quite expensive, increasing the required budget by 36–76%.
- Considering 1000 as the opening cost, the tabu search resulted in better solution both for robust and non-robust cases in one case out of five, therefore the last two rows are separate in order to differentiate the two approaches. The robust version of the problem could not be solved with the exact solver.
- For an extremely low opening cost, large number of facilities are opened and even the non-robust solution offer some robustness. However, the robustness can be improved relatively inexpensively (for $< 0.4$% of the budget) and in case of a disruption this can result in more than 10% saving in the additional costs.
- In each cases, either the local search or the tabu search could find the optimal solution for the uncapacitated facility location problem without robustness.

Table 2 contains the results of the solutions considering 100 locations. The following observations were made:

- For opening costs 5 million and 2.5 million local search and tabu search resulted in the same results as the exact solver. The non-robust solutions in these cases always contain only one open facility and adding robustness by opening more facilities are quite costly.
- For opening costs 1.6 and 1 million, the non-robust solutions contain one or two open facilities. The WCC and BoR values are the averages over the valid values. For these problems the solution of the local search and the tabu search often differ and it varies which performs better.
- For opening cost 1000, the tabu search performed better in one case. It can be observed that increasing robustness in this case is quite inexpensive, but the achieved benefit is also lower that in the 50 facility case.
- For only one problem instance neither the local search nor the tabu search heuristics could find the optimal solution for the uncapacitated facility location problem without robustness.

Table 3 contains the results of the solutions considering 500 locations. The following observations were made:

- With this size of solution space the result of the local search and the tabu search often differ and on average the tabu search performs slightly better.
- Most of the non-robust solutions contain two or more open facilities. Optimizing for robustness increases the cost usually under 20%, therefore as the problem size growths, it becomes relatively less expensive to provide robustness. However, in case of disruption, robust solutions can save at least 10% of the additional cost, when the facility opening cost is above one million.
- For five problem instances neither the local search, nor the tabu search heuristics could find the optimal solution for the uncapacitated facility location problem without robustness. Four out of these five cases have a low facility opening cost of 1000.

We conclude that robust solutions may save significant costs (in case of disruptions) especially when the number of opened facilities are low.

**Table 2:** Average performance using 100 locations

Opening	Non-robust			Robust					
	Open	Cost	WCC	Open	Cost	WCC	CoD (%)	PoR (%)	BoR (%)
5,000,000 (OPT)	1	7,579,528	–	2	12,383,253	12,594,382	1.72	63.58	–
5,000,000 (LS)	1	7,579,528	–	2	12,383,253	12,594,382	1.72	63.58	–
5,000,000 (TS)	1	7,579,528	–	2	12,383,253	12,594,382	1.72	63.58	–
2,500,000 (OPT)	1	5,079,528	–	2	7,383,253	7,594,382	2.90	45.67	–
2,500,000 (LS)	1	5,079,528	–	2	7,383,253	7,594,382	2.90	45.67	–
2,500,000 (TS)	1	5,079,528	–	2	7,383,253	7,594,382	2.90	45.67	–
1,666,666 (OPT)	1	4,246,195	–	2		5,927,715			
1,666,666 (LS)	1.2	4,327,338	8,102,164	2.2	5,703,829	6,083,148	6.33	32.85	5.87
1,666,666 (TS)	1	4,246,195	–	2	5,612,645	5,998,162	6.67	32.95	–
1,000,000 (OPT)	1.4	3,510,617		2		4,594,382			
1,000,000 (LS)	1.6	3,554,200	5,663,822	2.2	4,237,162	4,616,481	8.76	19.82	15.35
1,000,000 (TS)	1.4	3,533,251	6,062,458	2.2	4,242,211	4,628,458	8.85	20.56	17.01
1000 (OPT)	77.6	90,535	118,734						
1000 (LS)	77.6	90,535	118,734	78.2	90,703	113,882	25.67	0.18	4.05
1000 (TS)	77.6	90,535	118,734	78.6	90,829	113,859	25.49	0.32	4.07

**Table 3:** Average performance using 500 locations

Opening	Non-robust			Robust					
	Open	Cost	WCC	Open	Cost	WCC	CoD (%)	PoR (%)	BoR (%)
5,000,000 (OPT)	1.6	19,242,002							
5,000,000 (LS)	2	19,497,959	31,080,579	2.4	23,068,454	25,278,632	9.79	18.57	17.72
5,000,000 (TS)	1.6	19,307,357	33,319,075	2.4	23,062,160	25,421,665	10.31	19.78	20.13
2,500,000 (OPT)	2.4	14,263,693							
2,500,000 (LS)	2.2	14,372,353	25,267,673	3.8	16,318,181	19,023,002	17.26	13.69	22.33
2,500,000 (TS)	2.6	14,304,504	21,116,645	3.8	16,336,745	18,676,174	14.55	14.38	11.35
1,666,666 (OPT)	3.2	11,900,321							
1,666,666 (LS)	3	11,952,078	18,065,126	4	13,464,150	15,561,839	15.60	12.69	13.48
1,666,666 (TS)	3.4	11,905,914	17,393,652	4.8	13,380,271	15,323,262	14.60	12.36	11.43
1,000,000 (OPT)	4.4	9,496,951							
1,000,000 (LS)	4.4	9,496,951	13,729,015	6	10,402,176	11,881,160	14.26	9.60	12.61
1,000,000 (TS)	4.4	9,522,457	13,678,493	5.8	10,429,309	11,946,057	14.65	9.48	11.80
1000 (OPT)	282	396,016							
1000 (LS)	281.4	396,159	454,042	281.60	396,163	453,068	14.38	0.00	0.24
1000 (TS)	282	396,054	453,937	283	396,198	452,907	14.33	0.04	0.25

**Fig. 2:**
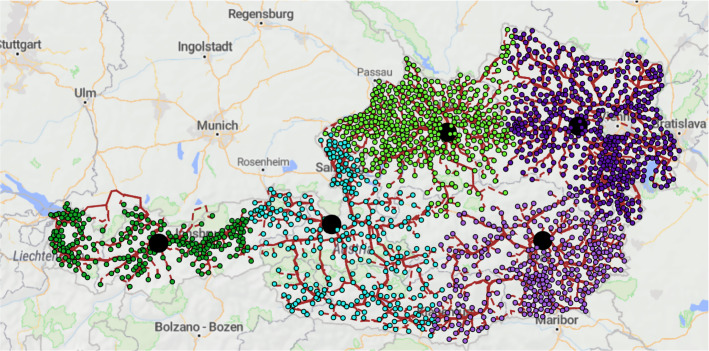
Robust waste wood collection network in Austria

We have also taken the whole dataset of 1839 accumulation points located in Austria and computed the location of the facilities in a robust network. Figure 2 illustrates the five open facilities (big black circles), the partitioning of the accumulation points (denoted with five different colors), as well as the routes used. The cost of operating the robust network is 45,710,341, which can increase up to 52,384,243 in case of a disruption—this is 14.6% additional transportation cost.

**Fig. 3:**
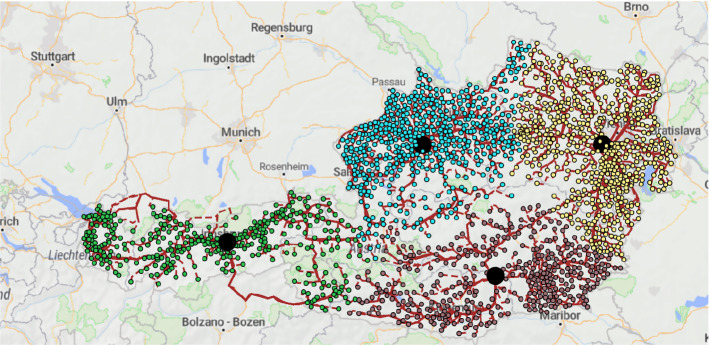
Non-robust waste wood collection network in Austria

Figure 3 shows the network in case robustness is not considered. The total cost in this case is 42,379,212, i.e., the robust solution is 7.86% more expensive. However, in case of a disruption the cost can grow by 29.65% to 54,943,410, i.e., it is 4.66% more than having a robust network.

### Numerical study on benchmark sets from the literature

Two different sets of benchmark data were used from the literature to further test our developed methods. The first set is a collection of artificial instances from the OR-Library (Beasley, 1990), one of the most widely used benchmark sets for uncapacitated facility location. The other set contains four real-world instances used by Buzna et al. (2014). They generated two of these real instances (road network of the Slovak Republic and the road network of six south-eastern U.S. states) based on available geographical data, while derived two instances of a Spanish road network from a dataset used in Dìaz and Fernández (2006).

Table 4 contains the results of the facility location for the OR-Library benchmark instances (the number in the brackets after the name of the instance indicates the number of accumulation points). The table contains only four columns, because for these instances the robust and non-robust solutions are the same, both using the local search and the tabu search heuristics. Table 5 contains the results of the remaining benchmarks, where either the robust solution differs from the non-robust one, or the two heuristics have different results. The exact solver could not determine the optimal solutions, not even for the small data sets.

**Table 4:** Benchmark sets

Data set	Open	Cost	WCC	CoD (%)
Cap71 (66)	16	4,004,139,523	4,426,181,538	10.54
Cap72 (66)	16	4,004,214,523	4,426,256,538	10.54
Cap73 (66)	16	4,004,289,523	4,426,331,538	10.54
Cap74 (66)	16	4,004,402,023	4,426,444,038	10.54
Cap101 (75)	25	2,860,332,101	3,092,624,089	8.12
Cap102 (75)	25	2,860,452,101	3,092,744,089	8.12
Cap103 (75)	25	2,860,572,101	3,092,864,089	8.12
Cap104 (75)	25	2,860,752,101	3,093,044,089	8.12
Cap131 (100)	47	2,850,307,905	3,037,867,617	6.58
Capc (1100)	70	227,277,815	233,771,069	2.86

**Table 5:** Benchmark sets

Data set	Non-robust			Robust					
	Open	Cost	WCC	Open	Cost	WCC	CoD (%)	PoR (%)	BoR (%)
Cap132 (100)	46	2,850,535,879	3,054,685,716	47	2,850,537,905	3,038,097,617	6.58	0.00	0.54
Cap133 (100)	46	2,850,760,879	3,054,910,716	47	2,850,767,905	3,038,327,617	6.58	0.00	0.54
Cap134 (100)	45	2,851,092,305	3,055,242,142	46	2,851,106,831	3,038,666,543	6.58	0.00	0.54
Capa (1100, LS)	43	314,947,367	330,044,167	43	314,947,367	330,044,167	4.79	0.00	0.00
Capa (1100, TS)	45	314,581,502	329,678,302	45	314,581,502	329,678,302	4.80	0.00	0.00
Capb (1100)	60	252,479,378	263,563,765	61	253,119,688	263,473,805	4.09	0.25	0.03

Table 6 contains the results of the experiments with the real benchmark data sets. Since the number of accumulation points is larger in these instances (indicated in brackets), only the results of the local search heuristic is presented. It can be observed that robust solutions contain more open facilities than non-robust ones, e.g., in case of the last dataset, the robust network consists of 6 more open facilities, which is more than 10% increase. However, the price of robustness is only around 2%, and the benefit is more than 6% in two of the four cases. It can also be noted that although the number of accumulation points is the largest in Slovak Republic, the number of suggested open facilities is much larger in the USA. This is due to the area of the countries: in the USA the points are more scattered, therefore more facilities are required to decrease the transportation costs.

**Table 6:** Real data sets

Data set	Non-robust			Robust					
	Open	Cost	WCC	Open	Cost	WCC	CoD (%)	PoR (%)	BoR (%)
Spain #1 (737)	27	374,471,197	414,065,300	31	377,588,678	389,076,109	3.04	0.83	6.04
Spain #2 (737)	10	134,325,907	150,149,399	13	137,200,214	145,576,082	6.10	2.14	3.05
Slovak Republic (2928)	15	204,267,943	225,709,011	16	207,024,319	222,589,817	7.52	1.35	1.38
USA (2398)	57	731,293,588	829,912,752	63	742,829,896	768,057,032	3.40	1.58	7.45

**Fig. 4:**
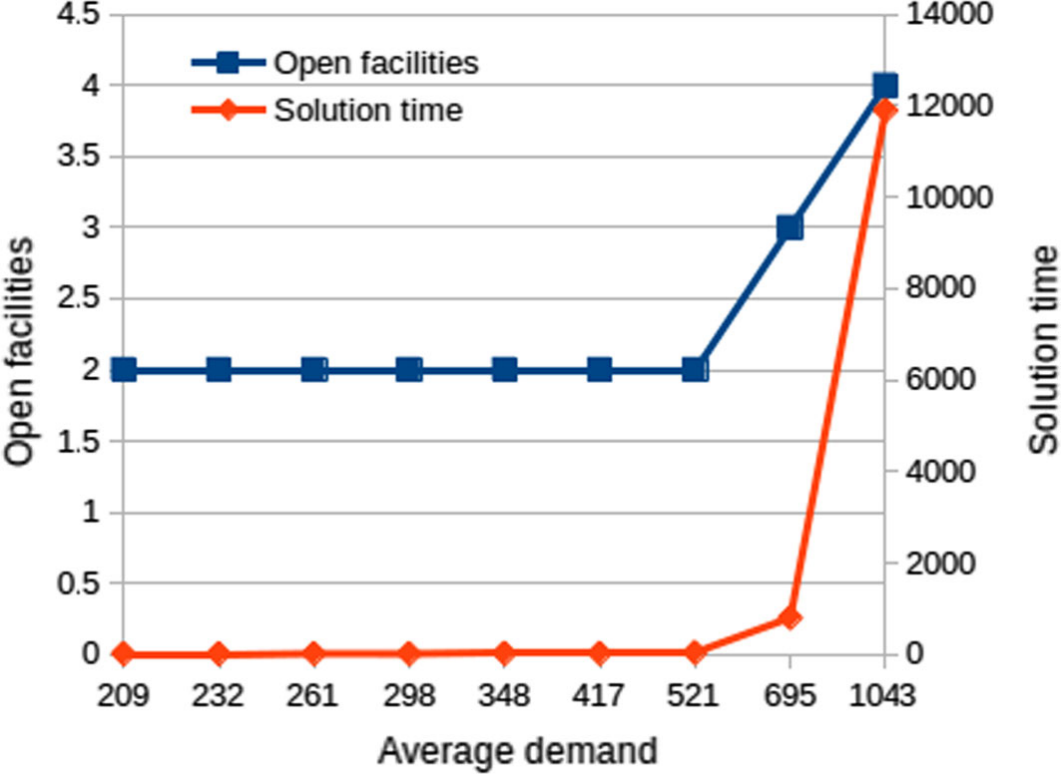
Solution time in seconds assuming different average demand

It is difficult to compare our algorithms with previous results, due to the differences in the model and in the datasets. However, additional experiments were conducted based on the facility location dataset provided in Daskin (2013), which is also the dataset used “with slight modifications” in the experiments of robust facility location by Cheng et al. (2021). In fact, in this latter paper the authors generated smaller subproblems consisting of different number of customers and potential facilities, while we used the whole dataset of 49 locations. Because of the differences in the examined and assumed datasets—huge demand, but relatively small facility opening costs, which is quite the opposite in the waste wood supply chain—the original problem could not be solved efficiently using the bilevel formulation. Instead of decreasing the problem size, we have multiplied the factory opening costs by 100 to approximate the typical costs of a wood recycling facility. Figure 4 shows the results of solving the bilevel formulation with assuming differently scaled demands. It can be seen that with small demands, low number of facilities are sufficient, and the optimal solution can be computed in approximately 15 s. As the demand—and thus the transportation costs—increases, more open facilities are necessary, and the computation time increases significantly.

### Numerical study of the economies of scale

In order to investigate the impact of the economies of scale, the test problems of Sect. 4.1 were also studied with different concave production costs using the objective function (6). Since the optimal number and locations of the facilities are based on the relation between three cost factors—the opening- the transportation- and the handling costs—, we have fixed the distance-based transportation cost in the following experiments and let the other two cost factors vary. Table 7 shows a typical result indicating the number of opened facilities resulted by different facility opening and production costs.

The specific example is based on a test set with 50 locations and the scale factor $b=-0.35$.

When $p_j=0$, the first row, the objective function is reduced to (1) instead of (6), i.e., the economies of scale is not considered. As expected, the number of opened facilities is inversely proportional with the opening cost. This can be observed in the rows of the table: with a fixed production cost, decreasing the opening cost results in the same number of open facilities or more. Similarly, considering a column with fixed opening cost, increasing the production cost results in fewer opened facilities.

The typical parameters of the waste wood industry (large facility opening cost and small production cost) can be found near the upper left corner of the table. With such parameters, the optimal network usually coincides with the optimal network without considering economies of scale: it is influenced mainly by the balance of the opening and transportation costs, while the effect of the production cost is negligible. Thus, we conclude that with typical cost parameters of the investigated industry, considering the economies of scale does not influence the solution significantly.

**Table 7:** Number of open facilities with economies of scale

Production cost ($p_j$)	Opening cost ($f_j$)						
	5,000,000	500,000	50,000	25,000	16,667	12,500	10,000
0	2	2	8	9	11	14	15
1	2	2	8	9	11	14	15
2	2	2	8	9	11	14	15
5	2	2	8	9	11	14	15
10	2	2	8	9	11	13	15
100	2	2	8	9	9	9	11
200	2	2	7	9	9	9	9
500	2	2	7	8	8	9	9
1000	2	2	5	5	7	8	8

## Conclusions

In this paper, we studied the facility location problem in the reverse logistics network of waste wood. This network considered the accumulation centre for waste wood as well as the processing facilities where they have to be transported. The traditional facility location was extended with the consideration of economies of scale and robustness against the breakdown of facilities. We formulated mathematical models for these problems including a novel approach based on bilevel optimization, and also presented a local and tabu search heuristic method for their solution.

We tested the efficiency of the proposed methods on instances generated using both a real-life industrial dataset and benchmark instances available in the literature. Different facility opening costs were considered, and robust and non-robust solutions were examined in every case. While economies of scale seemed to have no influence on the solutions in the case of realistic cost parameters, robustness turned out to be significant when the number of opened facilities was low. In the case of a larger number of opened facilities (which usually happened with unrealistically low facility costs) even the non-robust solutions contained some inherent robustness.

While the heuristic method gave the same solutions for instances with a smaller number of locations (where they mostly found the optimal solution), the tabu search had a slight edge over the local search for larger instances. However, we were not able to obtain exact solutions for these instances with a large number of locations, and working on mathematical programming methods to help the solution of the model will be part of our future work.

An important limitation of our model compared to other robust facility location models, is that we limit the possible number of disruptions to one. The reason behind this is that we are concerned with infrequent and random failures, instead of targeted attacks—which are not typical in the waste wood logistics. This limitation facilitates the novel bilevel integer program formulation, which can be solved more efficiently than general methods even for much larger problem instances, see e.g., (Cheng et al., 2021). However, it still cannot handle the huge networks typical in case of wood recycling. For these practical problems, we applied heuristics, and found that the tabu search using wider neighborhood in the medium term process yields significantly better results in reasonable time than frequency-based processes, e.g., Sun (2006).

As a future work, we intend to further study the integer program formulation of the bilevel robust facility location model and use it for computing lower bound on the cost. In addition, we are going to examine the delivery planning problem in the network designed by the facility location optimization.
